# New Approach for Designing Zinc Oxide Nanohybrids to Be Effective Photocatalysts for Water Purification in Sunlight

**DOI:** 10.3390/nano12122005

**Published:** 2022-06-10

**Authors:** Osama Saber, Aya Osama, Adil Alshoaibi, Nagih M. Shaalan, Doaa Osama

**Affiliations:** 1 Department of Physics, College of Science, King Faisal University, P.O. Box 400, Al-Ahsa 31982, Saudi Arabia; 217044956@student.kfu.edu.sa (A.O.); adshoaibi@kfu.edu.sa (A.A.); nmohammed@kfu.edu.sa (N.M.S.); 221445253@student.kfu.edu.sa (D.O.); 2 Petroleum Refining Department, Egyptian Petroleum Research Institute, Nasr City, P.O. Box 11727, Cairo 11765, Egypt; 3 Physics Department, Faculty of Science, Assiut University, Assiut 71516, Egypt

**Keywords:** inorganic-magnetic-organic nanohybrids, zinc oxide nanohybrid, water purification, sunlight

## Abstract

Water pollution and deficient energy are the main challenges for the scientific society across the world. In this trend, new approaches include designing zinc oxide nanohybrids to be very active in sunlight. In this line, organic and magnetic species intercalate among the nanolayers of Al/Zn to build inorganic-magnetic-organic nanohybrid structures. A series of nanolayered and nanohybrid structures have been prepared through intercalating very fine particles of cobalt iron oxide nanocomposites and long chains of organic fatty acids such as n-capric acid and stearic acid inside the nanolayered structures of Al/Zn. By thermal treatment, zinc oxide nanohybrids have been prepared and used for purifying water from colored pollutants using solar energy. The optical measurements have shown that the nanohybrid structure of zinc oxide leads to a clear reduction of band gap energy from 3.30 eV to 2.60 eV to be effective in sunlight. In this line, a complete removal of the colored pollutants (naphthol green B) was achieved after ten minutes in the presence of zinc oxide nanohybrid and sunlight. Finally, this new approach for designing photoactive nanohybrids leads to positive results for facing the energy- and water-related problems through using renewable and non-polluting energy for purifying water.

## 1. Introduction

The current situation of the water and energy in the world became more critical because of deficient energy and environmental problems. Two major challenges are clear for the scientific society in the recent years: water pollution and deficient energy. These international challenges are produced through the rapidly growing population and industries which led to these energy- and environment-related problems. Many scientists have used energy to solve the problem of water pollution leading to increasing the problem of deficient energy. For saving energy, the scientists tried to discover different techniques dependent on purifying water by renewable and non-polluting energy. One of the most familiar non-polluting resources for energy is sunlight. Solar energy can produce strong oxidizing agents for converting the industrial pollutants to carbon dioxide and water through exciting active photocatalysts. Most organic dyes such as textile dyes and surfactants are not easily biodegradable. Therefore, they belong to the colored hazardous pollutants. Photocatalytic degradation seems as one of the benign solutions for purifying water from organic dyes using photocatalysts and sunlight [1,2,3]. For solving these environmental problems, semiconductor photocatalysts are very familiar in this trend [4,5,6,7].

Although titanium oxide was one of the most famous photocatalysts in this field, their applications were limited because it can mainly absorb UV-light which considers 4% of the solar energy [8,9,10]. Therefore, zinc oxide is suggested to be an alternative photocatalyst to titanium oxide because it has large excitation binding energy of 60 meV, in addition to a band gap of 3.37 eV. According to the results of Dindar and Icli [11], zinc oxide was more effective than titanium oxide in sunlight for the degradation of phenol. Many researchers confirmed this conclusion through comparing between titanium oxide and zinc oxide semiconductors through the advanced oxidation of wastewater [12,13]. However, low performance of zinc oxide was observed for photocatalytic degradation in many studies [14,15,16] because of the high rate of recombination reactions for the excited electrons and holes of zinc oxide which happened within nanoseconds and the low amount energy absorbed during the photocatalytic processes. These disadvantages decrease the importance of photocatalytic degradation processes in the market.

Several techniques have been used for modifying the structure of zinc oxide to solve its problems through narrowing its band gap energy to be active in sunlight. The formation of nanostructures [17], in combination with carbon nanorods and nanotubes [18], and introducing surface defects, were good solutions for improving the activity of zinc oxides. Additionally, for preventing the disadvantages of zinc oxide, doping processes with transition elements in addition to morphological changes [19] were studied to be suitable solutions for increasing the performance of zinc oxide for the photocatalytic degradation of pollutants. In this trend, the optical properties and activity of zinc oxide were developed through the morphological changes from nanoparticles [20] to nanorods [21]. In addition, the zinc oxides nanotubes [22], and nanowires [23] were suggested to be active photocatalysts [24].

Many researchers have used transition elements for doping zinc oxide to become effective photocatalysts [25,26,27,28,29]. Insertion of sulfur inside the structure of ZnO improved the charges separation through preventing the recombination process between electrons and holes [30]. The results of Adeel et al. showed high photocatalytic degradation of rhodamine blue and methylene blue under UV irradiation using ZnO films which were modified by the addition of Ag and Al [31]. The introduction of nitrogen using micro-emulsion method increased the optical properties and activity of ZnO nanospheres [32]. In addition, several studies concluded that the addition of aluminum and iron as dopants inside ZnO structures converted their transparent thin films to be useful for photocatalytic applications and solar cells [33,34,35,36,37,38]. This positive effect of the addition of aluminum inside zinc oxide was confirmed by our previous research [7]. Thus, the current study concentrates on improving the photocatalytic performance of ZnO structures through building nanohybrids based on organic, magnetic, and inorganic species by an unconventional technique. In the conventional methods [39], multi-steps were used for mixing one or two elements for zinc oxides. However, the main challenge is to get a homogenous structure of all elements inside the crystals of ZnO.

The nano-size spinel ferrite nanoparticles CoFe_2_O_4_ have recently attracted significant attention because of their remarkable photocatalytic properties [40,41,42,43]. Although much research has been carried out for studying the photocatalytic performance of cobalt ferrite nanoparticles, there are no articles for using CoFe_2_O_4_ as a filler for zinc oxide structures. Additionally, because of the low band gap energy of cobalt iron oxide CoFe_2_O_4_ (1.32 eV) [43], it is considered an excellent dopant and filler for reducing the band gap energy for zinc oxide.

Following this trend, the current study has used a new strategy for building zinc oxide nanohybrids with reduction in the optical band gap energy to be active in visible light for purifying water from pollutants. In this strategy, a series of zinc oxide nanohybrids based on magnetic, inorganic, and organic species were prepared through building inorganic-magnetic-organic nanohybrids according to the host–guest interaction. The inorganic-magnetic-organic nanohybrids were formed by intercalation reactions of long chains of hydrocarbons of n-capric acid and inserting magnetic nanoparticles of cobalt iron oxides nanocomposites inside the nanolayered structures of zinc and aluminum. Furthermore, longer chains of organic acid (stearic acid) were used for studying the role and the effect of organic species. Organic species are used as pillars to widen the interlayered spacing of the nanolayered structures to allow for the magnetic nanoparticles to be inserted between the nanolayers of zinc and aluminum. These nanohybrids were used for producing zinc oxide nanohybrids by thermal treatment. Zinc oxide nanohybrids were tested for purifying the water using sunlight through photocatalytic degradation of the colored pollutants. At the same time, the optical properties and activity of the nanohybrids were studied and compared with conventional photocatalysts. This strategy depends on a good distribution of magnetic nanocomposites inside the internal surfaces of the Al/Zn nanolayers in special arrangements in the nano scale producing nanohybrids based on zinc oxide doped with multi-oxides, aiming to creating new optical active centers.

## 2. Materials and Methods

The hetero-structured hybrids such as inorganic-magnetic-organic systems are good candidates for creating a distinguished photo-activity for ZnO which cannot be achieved through the usual methods. In order to build inorganic-magnetic-organic nanohybrids based on zinc oxide, three types of nanomaterials were prepared. The first type was very fine nanoparticles of cobalt iron oxides nanocomposite, which were used as filler for the nanohybrids. The second one was nanolayered structures based on Al/Zn LDHs. The third type depended on the long chains of hydrocarbon of organic fatty acid to expand and widen the interlayered spacing of the nanolayered structures. This widening and expansion can facilitate the insertion of magnetic nanoparticles among the nanolayers of Al/Zn LDHs. To study the important role of organic fatty acid, two different kinds of long chains of hydrocarbons were used for building the nanohybrid structures.

### 2.1. Preparation of Nanoparticles of Magnetic Nanocomposites

A solvent thermal technique has been used for preparing very fine nanoparticles of cobalt iron nanocomposite. Cobalt (II) acetate (0.051 mol) and Iron (II) acetate (0.026 mol) were reacted with 350 mL of methanol at room temperature for 5 h. A similar amount of ethanol was added to the mixture. To complete the reaction under super critical conditions of pressure and temperature, the mixture was placed inside an autoclave. The mixture was heated by a slow rate of 1 °C/min. to reach 260 °C under a high pressure of 75 bar. At this stage, the pressure was slowly released under a flow of nitrogen to avoid oxidation reactions. At the same time, the temperature of the autoclave decreased to the room temperature. The fine powder of the product was easily collected.

### 2.2. Preparation of Nanolayered Structures and Nanohybrids

Two samples of inorganic-magnetic-organic nanohybrids were prepared for designing nanolayered structure with changing the organic species. The pure nanolayered structure of Al/Zn LDH was prepared without magnetic or organic species for comparison. Al/Zn LDH was prepared through mixing aqueous solutions (0.069 M) of aluminum nitrate with zinc nitrate in presence of 0.5 M of urea. The molar ratio of aluminum to zinc was 1:3. By keeping the temperature of the mixture at 80 °C, the nanolayers of LDH were precipitated during the hydrolysis of urea because the nature of reaction medium were gradually changed from acidic to alkaline. White precipitate was obtained after 12 h of the reaction. It was filtrated and washed by distilled water. By drying at room temperature, the product was collected and coded by ZOA.

The inorganic-magnetic-organic nanohybrid was synthesized by adding 100 mL of the aqueous solution of 5% n-capric acid sodium salt during building the Al/Zn nanolayered structure. In addition, 0.5 g of the prepared nanoparticles of cobalt iron oxides nanocomposite was mixed with the aqueous solution (0.069 M) of aluminum nitrate with zinc nitrate in presence of 0.5 M of urea. By keeping the temperature of the mixture at 80 °C, the product was obtained after 12 h of the reaction. After filtration and washing, the sample was dried at 25 °C in vacuum box. The product was coded as HZ-1.

Another inorganic-magnetic-organic nanohybrid was prepared by the same procedure by changing the organic compound n-capric acid to be stearic acid, noting that the nanolayers of the Al/Zn LDH were precipitated in presence of 0.5 g of the prepared nanoparticles of cobalt iron oxides nanocomposite with the organic species. The product was collected and coded as HZ-2.

### 2.3. Preparation of Nanohybridsand Nanocomposite Based on Oxides

The nanolayered structure of Al/Zn LDH was thermally treated at 500 °C to produce a nanocomposite of zinc and aluminum oxides. It was represented by ZOA-500. By calcination at 500 °C, the nanohybrid HZ-1 was converted to be a stable nanohybrid composed of magnetic and non-magnetic oxides. It was represented as HZ-1-500. The inorganic-magnetic-organic nanohybrid HZ-2 was transformed to new structure of nanohybrid through the thermal treatment at 500 °C. It was represented as HZ-2-500.

### 2.4. Physical Characterization

Nanolayered structures and crystalline structures of the prepared samples were identified by a Bruker-AXS system with Cu-Ka radiation (Bruker Company, Karlsruhe, Germany) for X-ray diffraction analysis (XRD). An electrons probe microanalyser JED2300 (JEOLCompany, Tokyo, Japan) was used for detecting the different metals in the products through energy dispersive X-ray spectroscopy (EDX). For studying the thermal behavior of the prepared samples, a thermogravimetric analyzer TA series Q500 and TA series Q600 for differential scanning calorimetry (DSC) (T-A company, New Castle, PA, USA) were used under the flow of nitrogen. FTIR spectroscopy was performed by using a Perkin–Elmer Spectrum 400 instrument starting from 425 cm^−1^ to 4000 cm^−1^. For determining the morphology and nanosize of the products, scanning electron microscopy (SEM) and transmission electron microscopy (TEM) JEM 2100F (JEOL Company, Tokyo, Japan) was used with different magnifications. The optical properties were measured for the prepared samples through the diffuse reflectance technique. UV/VIS/NIR Shimadzu 3600 spectrophotometer (Shimadzu, Columbia, MD, USA) was used for measuring the absorbance of liquid and solid samples.

### 2.5. Photocatalytic Activity

Photocatalytic degradation of aqueous solutions of industrial dyes was used for measuring the photocatalytic activity of the prepared materials for purification of water in sunlight. The photo activities of the prepared nanomaterials were studied through photocatalytic reactions of the green dyes such as naphthol green B (NGB) in the sunlight. In the current research, the low concentration of aqueous solution of NGB (4 × 10^−4^ M) was prepared and mixed with 0.1 g of the one of prepared nanomaterial. This sample was exposed to sunlight through an irradiation area of 10 cm^2^. According to the Beer-Lambert law, the intensity of the measured spectrum of the dye can be used for expressing the low concentration of the dye. Depending on this fact, a small quantity of the green solution was extracted from the main mixture every few minutes during exposure to sunlight. Then, the UV-Vis spectrophotometer can determine the concentration through calculating the intensity of absorbance of the liquid samples at 714 nm, which is the characteristic peak of NGB. During the spring season (March) in Saudi Arabia, the photocatalysis processes were carried out in presence of sunlight starting from 10:00 a.m. to 10:20 a.m.

## 3. Results

### 3.1. Characterization of the Prepared Filler

**Figure 1:**
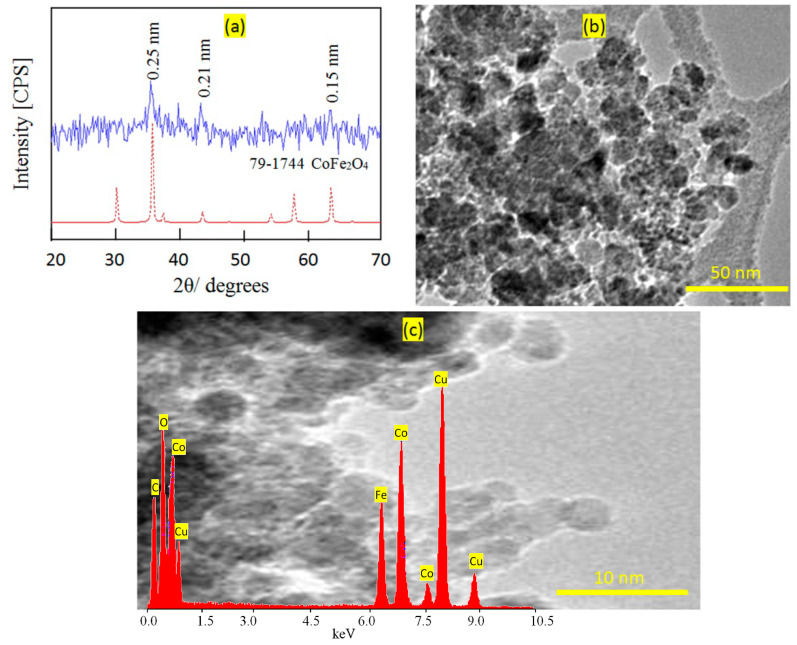
The prepared cobalt iron oxides nanocomposite (**a**) X-ray diffraction pattern (the red line is the standard and the blue line is the sample), (**b**) TEM image at 50 nm, and (**c**) TEM image at 10 nm (Inset: EDX spectrum).

Very fine nanoparticles of cobalt iron oxides nanocomposite were prepared and characterized to be suitable for use as a filler and inserted among the nanolayers of the nanolayered structures. In this trend, X-ray diffraction was used for confirming the structure of the prepared cobalt iron oxides nanocomposite. Figure 1a shows the X-ray diffraction pattern of the prepared cobalt iron oxides nanocomposite.

The X-ray diffraction pattern showed weak peaks at 2Ѳ = 35.56°, 41.6°, and 62.9°, agreeing with d-spacings at 0.25 nm, 0.21 nm, and 0.15 nm, respectively. According to the diffraction lines of JCPDS 79–1744, Figure 1a reveals that the prepared cobalt iron oxides have a CoFe_2_O_4_ structure. Transmission electron microscopy was used for measuring the nano size of the particles of the prepared cobalt iron oxides. Figure 1b shows strong aggregates of nanoparticles because of the magnetic behavior of the cobalt iron oxides. By magnification, very fine nanoparticles are observed in Figure 1c. Figure 1c shows that the size of the particles of the prepared cobalt iron oxides is less than 5 nm. Energy dispersive X-ray spectrometry (EDX) analysis confirmed the presence of magnetic elements through observing two sharp peaks for cobalt and iron, as shown in Figure 1c (inset).

### 3.2. Design of Inorganic-Magnetic-Organic Nanohybrids

**Figure 2:**
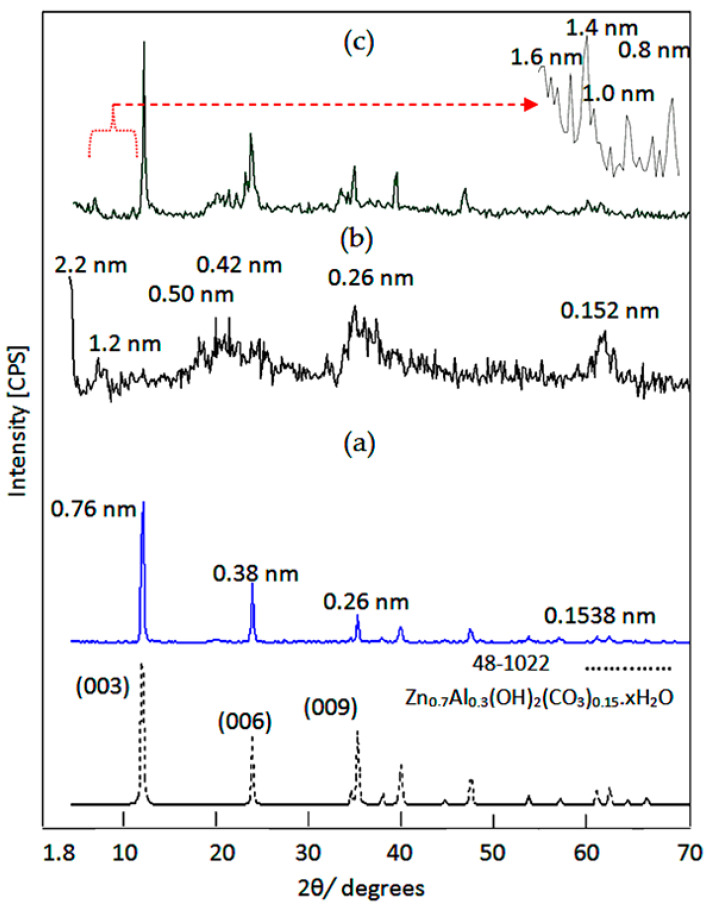
X-ray diffraction patterns of: (**a**) the pure Al/Zn nanolayered structure, (**b**) the nanohybrid HZ-1, and (**c**) the nanohybrid HZ-2.

Inorganic-magnetic-organic nanohybrids appear to be very creative because they can produce unlimited sets of known or unknown properties. In this way, nanohybrids were designed by a combination of zero-dimensional nanoparticles of magnetic nanocomposite and two-dimensional nanolayered structures, in addition to long chains of organic acid. This combination was achieved in an order arrangement through building Al/Zn nanolayered structures which have cationic nanolayers. In the presence of n-capric acid (CH_3_(CH_2_)_8_COO^−^), the long chains of the aliphatic acid were intercalated among the nanolayers for neutralizing their positive charges. At the same time, the long chains of hydrocarbons of organic compounds were working as pillars for building the nanolayered structures. In addition, these pillars expanded and widened the interlayered spacing among the nanolayers to produce enough space for existing magnetic nanoparticles of cobalt iron oxides nanocomposite. To indicate the positive role of organic species for designing this nanohybrid, a pure Al/Zn nanolayered structure was prepared for comparison. In addition, the Al/Zn nanolayered structure was modified by the nanoparticles of cobalt iron oxides nanocomposite with longer chains of hydrocarbon of stearic acid to study the effect of the organic species. The X-ray diffraction patterns of the prepared nanolayered structures and nanohybrids are displayed in Figure 2.

Figure 2a shows the x-ray diffraction pattern of the pure Al/Zn nanolayered structure. Sharp and symmetric peaks were observed at 2Ѳ = 11.62°, 23.36°, and 34.54°, aligning with the d-spacing of 0.76 nm, 0.38 nm, and 0.26 nm, respectively. These peaks are due to the reflections of the main planes (003), (006), and (009). The clear arrangement between these reflections (0.76 nm = 2 × 0.38 nm = 3 × 0.26 nm) confirmed formation of the nanolayered structures of the natural hydrotalcite (JCPDS file No. 37–629) and zinc aluminum carbonate hydroxide hydrate (JCPDS file No. 38–486). The other reflections of the planes (012), (015), (110), and (113) of the nanolayered structures of the natural hydrotalcite were observed at 2Ѳ = 39.16°, 46.56°, 60.05°, and 61.44° and matched with the d-spacing of 0.23 nm, 0.19 nm, 0.17 nm, 153, and 0.150 nm, respectively. The crystal parameters (a, c) could be calculated depending on the d-spacing of the planes (003) and (110), respectively. The first parameter was 2 × d_(110)_ = 0.306 nm. It means that the average distance between Zn-cation and Al-cation is 0.306 nm, agreeing with the previous published data of zinc aluminum carbonate hydroxide hydrate (JCPDS file No. 38–486). The second parameter was assessed by 3 × d_(003)_ = 2.28 nm. It was similar to that reported for the natural hydrotalcite.

By intercalating the long chains of hydrocarbons of n-capric acid (CH_3_(CH_2_)_8_COO^−^) with the Al/Zn nanolayered structures in presence of the nanoparticles of cobalt iron oxides nanocomposite, inorganic-magnetic-organic nanohybrid HZ-1 was formed through a host–guest interaction. The X-ray diffraction pattern of HZ-1, which is displayed in Figure 2b, shows new peaks at low 2Ѳ in addition to the disappearance of the original peaks of the nanolayered structure of LDH, noting that the peaks of the nanoparticles of magnetic nanocomposite were observed as weak peaks at 2Ѳ = 35.56°, 41.6°, and 62.9°. A sharp peak was observed at 2.2 nm indicating that the interlayered spacing of the nanolayered structure expanded and widened from 0.755 nm to 2.20 nm. This spacing could allow for the nanoparticles of cobalt iron oxides to intercalate among the nanolayers of the nanolayered structure because the peaks of cobalt iron oxides were not clear in Figure 2b. The crystal parameter (a), which depends on the reflection of the plane (110), has a little shift. At the same time, a large change was observed for the parameter (c) from 2.280 nm to 6.60 nm. It means that the nanohybrid HZ-1 consists of nanolayered structures having organic species and magnetic nanoparticles.

**Figure 3:**
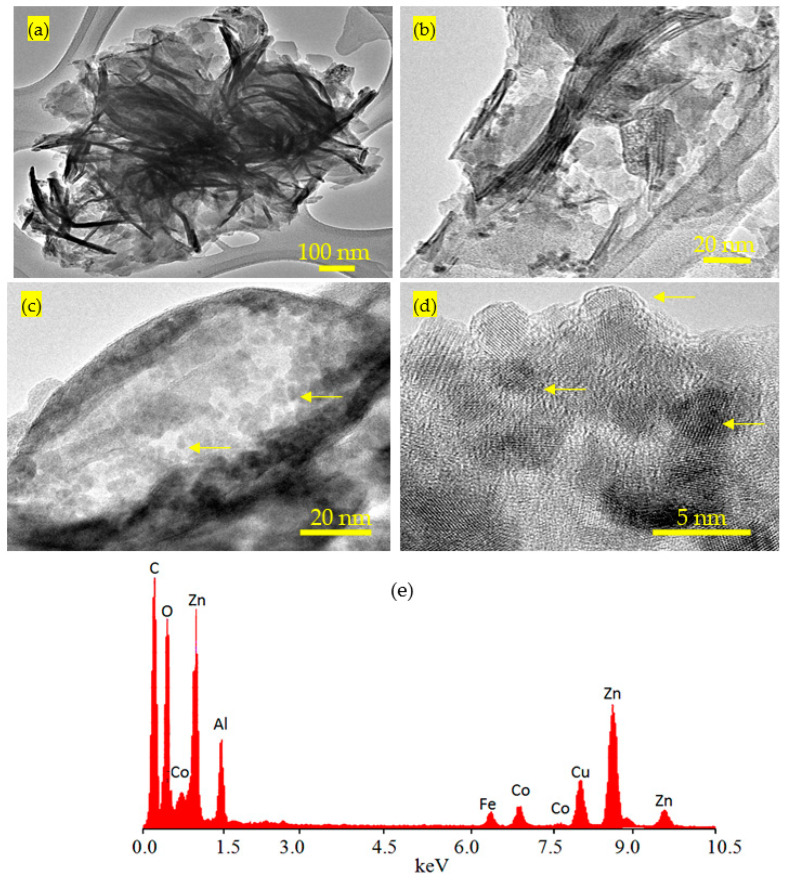
TEM images of the nanohybrid HZ-1: (**a**) at 100 nm, (**b**,**c**) at 20 nm, (**d**) at 5 nm (the yellow arrows are due to the intercalated nanoparticles), and (**e**) EDX spectrum.

With intercalating longer chains of organic compounds, stearic acid (CH_3_(CH_2_)_16_COO^−^) and the nanoparticles of cobalt iron oxides nanocomposite inside the pure Al/Zn nanolayered structures, HZ-2 was formed to build another inorganic-magnetic-organic nanohybrid. Figure 2c shows the main peaks of the Al/Zn LDH in addition to appearing as new peaks after building the nanohybrid HZ-2. The new peaks of the nanohybrid HZ-2 were observed at 1.6 nm and 1.4 nm, as seen in Figure 2c (inset). It indicated that the interlayered spacing of the nanolayered structure expanded and widened from 0.755 nm to become higher. This expansion could allow for the nanoparticles of cobalt iron oxides to intercalate among the nanolayers of the nanolayered structure because the characteristic peak of cobalt iron oxides overlaps with the peak of nanolayered structure at 2Ѳ = 35.56°, as shown in Figure 3c. It means that the nanohybrid HZ-2 consists of nanolayered structures that have organic species and magnetic nanoparticles.

This finding was confirmed by transmission electron microscopy (TEM) and energy dispersive X-ray spectrometry (EDX). TEM images of the nanohybrid HZ-1 are displayed in Figure 3. Figure 3a shows that the nanohybrid HZ-2 has nanoplatelets with a size of less than 50 nm. Furthermore, very fine nanoparticles, which are marked by arrows, were observed in Figure 3c,d, representing the magnetic nanoparticles cobalt iron oxides. Through magnification, Figure 3c confirmed the presence of the magnetic nanoparticles among the nanolayers of nanohybrid. In addition, Figure 3d shows one particle started to intercalate with the nanolayered structure. By EDX analysis, the different elements were identified in the nanohybrid HZ-2, as shown in Figure 3e. Figure 3e shows sharp peaks for the non-magnetic elements zinc and aluminum. In addition, the magnetic elements cobalt and iron were observed by weak peaks.

**Figure 4:**
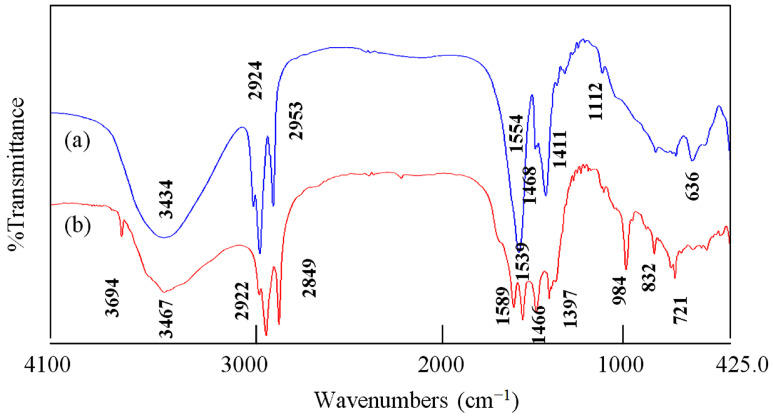
FT-IR spectra of: (**a**) the nanohybrid HZ-1 and (**b**) the nanohybrid HZ-2.

**Figure 5:**
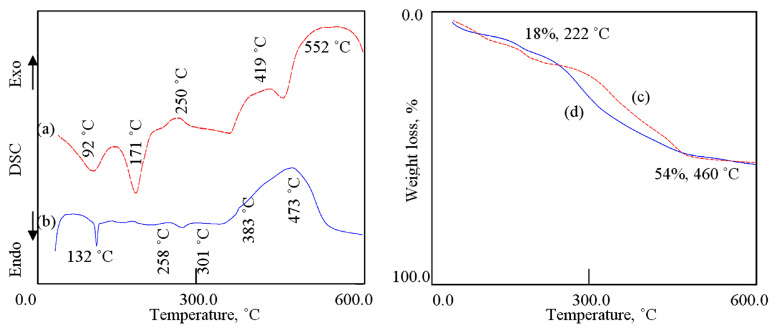
Thermal analyses of: (**a**) DSC curve of HZ-1, (**b**) DSC curve of HZ-2, (**c**) TG curve of HZ-1, and (**d**) TG curve of HZ-2.

In order to identify the function groups of the nanohybrids HZ-1 and HZ-2, the infrared spectra (FT-IR) was used and is displayed in Figure 4. For the nanohybrid HZ-1, the absorption band was observed at 3434 cm^−1^, indicating the stretching mode of hydroxyl groups as seen in Figure 5a. The presence of long chains of hydrocarbon of n-capric acid was clear in the IR spectrum because the stretch absorption of carbon–hydrogen was observed by sharp peaks at 2924 cm^−1^ and 2953 cm^−1^. In addition, the bending mode of the carbon–hydrogen was clear through an observing band at 1468 cm^−1^. The symmetric stretching vibration of carboxylate, which belonged to the aliphatic acid, was observed at 1554 cm^−1^. Furthermore, the absorption at 1411 cm^−1^ is assigned to the asymmetric stretching vibration of carboxylate. The bands observed below 1000 cm^−1^ could be ascribed to Zn-O and Al-O.

For the nanohybrid HZ-2, Figure 4b confirms the formation of inorganic-magnetic-organic nanohybrid through observing the main bands of stearic acid. The presence of long chains of hydrocarbon was confirmed by observing sharp peaks at 2922 cm^−1^ and 2849 cm^−1^, indicating the stretch absorption of carbon–hydrogen. Additionally, the bending mode of the carbon–hydrogen was clear through observing the band at 1466 cm^−1^. The symmetric stretching vibration of carboxylate, which belonged to the aliphatic acid, was observed at 1589 cm^−1^. Furthermore, the absorption at 1397 cm^−1^ is assigned to the asymmetric stretching vibration of carboxylate. In addition, the absorption band of the hydroxyl groups of the nanolayered structure was observed at 3467 cm^−1^. In the same trend, the presence of different kinds of hydroxyl groups were confirmed by observing another band for hydroxyl groups at 3694 cm^−1^. It indicated that the presence of the nanoparticles of cobalt iron oxides among the nanolayers affect the vibrational mode of hydroxyl groups. It means that the confinement of the nanoparticles of CoFe_2_O_4_ among the nanolayers affect the hydroxyl groups which are closer to these nanoparticles.

The thermal gravimetric analysis and differential scanning calorimetry (TGA-DSC curves) were used to study the thermal behavior of the prepared nanohybrids. Figure 5 indicates that the thermal decomposition of both HZ-1 and HZ-2 can give information for the nature of the interlayer species inside the nanohybrids. The DSC curve of the nanohybrid HZ-1 shows two series of peaks, as shown in Figure 5a. The first series is endothermic peaks at 92 °C and 171 °C, which are ascribed to the removal of the surface and interlayered water. The second series is exothermic peaks at 250 °C, 419 °C, and 552 °C, representing the oxidation reactions of the chains of hydrocarbon of n-capric acid. From the TG curve (Figure 5c), the weight loss of 18%, which happened up to 222 °C, represents the internal content of water inside the nanohybrid HZ-1. In the same way, the weight loss of 36%, which occurred up to 460 °C, is due to the internal content of organic species inside the nanohybrid HZ-1. The DSC curve of the nanohybrid NHA-2 is similar to that of the nanohybrid HZ-2, as seen in Figure 5b. Figure 5b shows endothermic and exothermic peaks, indicating the removal of water and oxidation reactions of the long chains of hydrocarbon of stearic acid. In the same way, similar behavior was observed for the TG curve of HZ-2, as shown in Figure 5d. The thermal analyses results confirmed formation of the nanohybrids HZ-1 and HZ-2.

### 3.3. Design of Nanohybrids Based on Oxides

The main reason for designing nanohybrids with organic and inorganic species is directed to produce stable and effective zinc oxides nanohybrids and nanocomposites with distinguished properties. Therefore, the prepared nanohybrids were thermally treated at 500 °C to remove unstable species and create new active sites.

**Figure 6:**
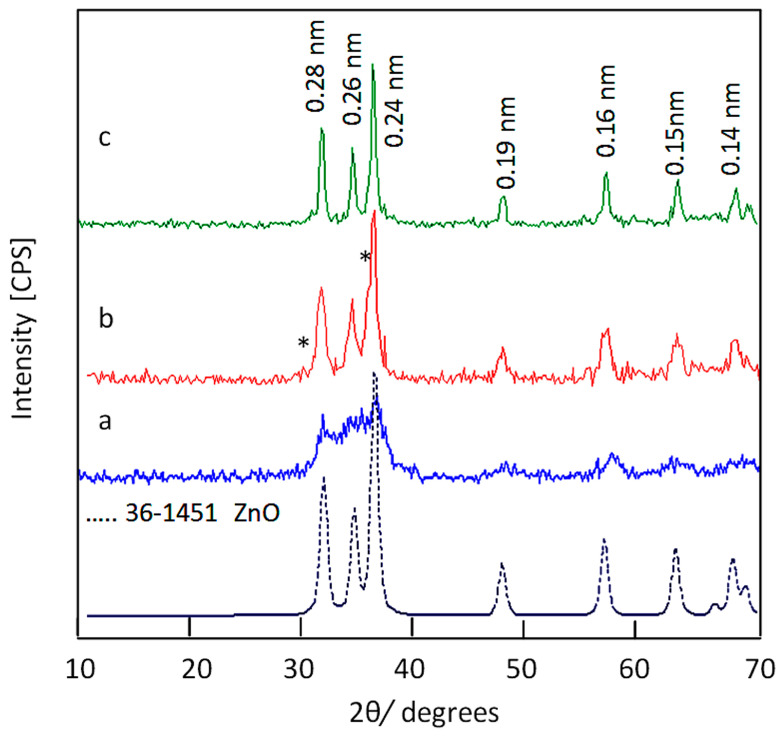
X-ray diffraction patterns of: (**a**) ZOA-500, (**b**) HZ-1-500 (the * is due to cobalt iron oxides), and (**c**) HZ-2-500.

X-ray diffraction has been used to identify the produced structures from the calcination of the nanohybrids. Figure 6 shows X-ray diffraction patterns of ZOA-500, HZ-1-500, and HZ-2-500. The XRD pattern of ZOA-500 exhibited new weak peaks at 2Ѳ = 32.01°, 34.32°, 36.49°, 47.71°, 7.05°, and 62.81°, in addition to disappearance of the original peaks of the nanolayered structures, as shown in Figure 6a. By comparing the diffraction lines of the zinc oxide crystal (JCPDS No. 36-1451) and the standard entire diffraction pattern of zincite phase (JCPDS No. 75-576), ZOA-500 has a similar structure for zinc oxide. However, the broad and diffuse peaks of ZOA-500 indicated that the structure of ZOA-500 is not pure because of the presence of the amorphous structure of aluminum oxide inside the zincite phase. In case of the nanohybrid HZ-1-500, Figure 6b shows clear and sharp peaks at 0.28 nm, 0.26 nm, and 0.24 nm, indicating a crystalline structure. Furthermore, weak peaks were observed at 0.19 nm, 0.16 nm, 0.15 nm, and 0.14 nm. These diffraction lines agree with the peaks of the zinc oxide crystal (JCPDS No. 36-1451) and the standard entire diffraction pattern of the zincite phase (JCPDS No. 75-576). In addition, a weak peak was observed at 0.30 nm and marked with (*) in Figure 6b. At the same time, Figure 6b reveals that the characteristic peak of cobalt iron oxides is observed at 0.25 nm and overlaps with the peak of zinc oxide at 0.24 nm.

For the nanohybrid HZ-2-500, Figure 6c shows that the characteristic peaks of zinc oxide were observed at 0.28 nm, 0.26 nm, and 0.24 nm, agreeing with the crystalline structure of the sample HZ-1-500. This similarity was confirmed by observing weak peaks at 0.19 nm, 0.16 nm, 0.15 nm, and 0.14 nm. These diffraction lines agree with the peaks of the zinc oxide crystal (JCPDS No. 36-1451) and the standard entire diffraction pattern of zincite phase (JCPDS No. 75-576). At the same time, Figure 6c revealed that the characteristic peak of cobalt iron oxides at 0.25 nm were not clear in the sample NH-2-500. These XRD results can conclude that both HZ-1-500 and HZ-2-500 have a zincite phase doping with aluminum and cobalt iron oxides.

**Figure 7:**
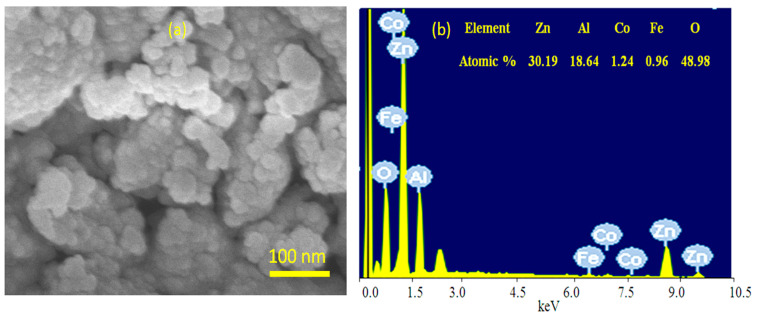
Images and spectrum of HZ-1-500: (**a**) SEM and (**b**) EDX with chemical composition.

To confirm the presence of magnetic elements inside the ZnO crystals, the chemical composition of HZ-1-500 was measured through scanning electron microscopy (SEM) and energy dispersive X-ray spectrometry (EDX). SEM images showed that HZ-1-500 has one phase, as shown in Figure 7a. In addition, SEM image indicated that this phase consisted of nanoparticles. The chemical composition of this phase was determined by the EDX equipment, which is attached to SEM. The EDX spectrum confirmed the presence of magnetic elements Co and Fe. Furthermore, Figure 7b shows that the atomic percentages of cobalt and iron in the nanoparticles of HZ-1-500 are 1.24% and 0.96%; respectively. In addition, the atomic percentage of aluminum was 18.64%. At the same time, Figure 7b revealed that the highest percentage is due to zinc. It means that HZ-1-500 is composed of zinc oxide doping with Al, Co, and Fe.

**Figure 8:**
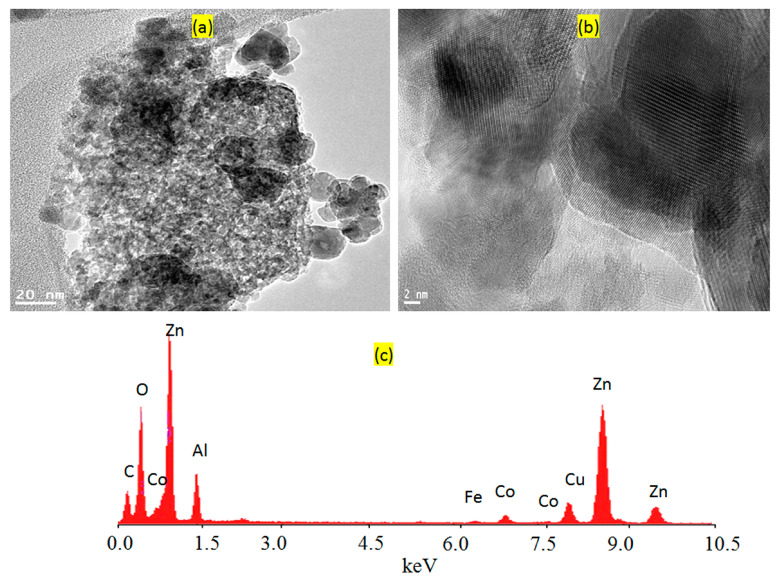
TEM images and EDX of HZ-1-500: (**a**) 20 nm, (**b**) 2 nm, and (**c**) EDX spectrum.

TEM images of HZ-2-500 confirmed this finding, as shown in Figure 8. Clear nanoparticles were observed for HZ-1-500, as seen in Figure 8a. It indicated that the width of HZ-1-500 is 20 nm. Additionally, very fine white spots were observed and marked by the arrow on the surface of the nanoparticles. These spots represent the cobalt iron oxides nanocomposites. These white spots became clearer by magnification, as seen in Figure 8b. Figure 8b shows the combination between the zinc oxide particles with the particles of cobalt iron oxides. Energy dispersive X-ray spectrometry (EDX) analysis of HZ-1-500 confirmed the presence of magnetic elements through observing two weak peaks for cobalt and iron, as seen in Figure 8c. Furthermore, inorganic elements (zinc, aluminum and oxygen) were also observed by sharp peaks in Figure 8c.

**Figure 9:**
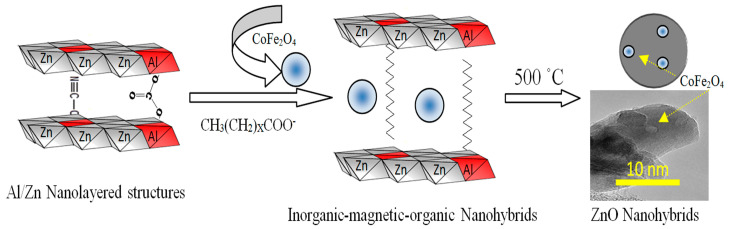
Schematic representation of zinc oxide nanohybrids based on inorganic-magnetic-organic nanohybrids.

According to the results of XRD and the images of TEM in addition to SEM-EDX analysis, the zinc oxide nanohybrids were produced from the thermal decomposition of the inorganic-magnetic-organic nanohybrid, as shown in Figure 9.

Figure 9 shows a schematic representation for transforming the inorganic-magnetic-organic nanohybrid to zinc oxides nanohybrids. It indicates that the presence of the magnetic nanoparticles of cobalt iron oxides nanocomposite among the nanolayers of Al/Zn gave a good chance for incorporation of cobalt iron oxides nanocomposite with the nanoparticles of the Al-doped ZnO, which was produced during the thermal decomposition of organic species. This combination, which happened during the crystallization process of zinc oxide, created new optical active sites for HZ-1-500. According to the similarity between the results of XRD, FIIR, and thermal analyses of both nanohybrids HZ-1 and HZ-2, a similar process happened to produce HZ-2-500.

### 3.4. Optical Properties

Zinc oxide is familiar for the researchers in the field of optical application as one of the most famous photo-active materials. However, its optical applications are concentrated in the UV-region. Therefore, many studies were published in literature for developing the structure and the morphology of zinc oxide to advance its optical behavior through increasing the range of its absorbance and decreasing its band gap energy

**Figure 10:**
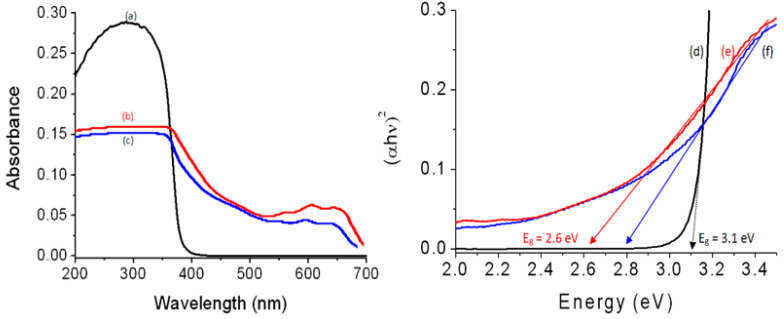
UV-Vis absorbance of: (**a**) ZOA-500, (**b**) HZ-1-500, (**c**) HZ-2-500, and band gap energy of (**d**) ZOA-500, (**e**) HZ-1-500, and (**f**) HZ-2-500.

In this way, the optical absorbance and the band gap energy of the prepared nanohybrids were studied and compared by using the UV-Vis absorption technique which considers one of the main means for giving significant details about their optical properties. Figure 10 shows the UV-Vis absorbance of ZOA-500, HZ-1-500, and HZ-2-500 in addition to their band gap energy. Figure 10a indicates that ZOA-500 is active in the UV region because it has absorption in the range of wavelengths of 200–350 nm. At the same time, there is no absorption in the visible region above 400 nm. By modifying the structure of ZOA-500 through building nanohybrid with magnetic nanocomposites and n-capric acid, the optical properties of HZ-1-500 improved, as shown in Figure 10b. A new absorbance band was observed in the visible region at 630 nm. At the same time, the absorbance edge shifted to higher wavelength at 700 nm. This positive effect was also observed for HZ-2-500, as shown in Figure 10c. Figure 10c shows clear absorbance for HZ-2-500, starting from 700 nm to 200 nm with two maxima at 600 nm and 350 nm. It means that the intercalation of magnetic nanoparticles inside the interlayered space of the nanohybrid led to good and ordered dispersion inside the structure of zinc oxide, in addition to creating new optical active centers for ZnO after calcination.

These results were confirmed by calculating their optical band gap energies. The band gap energy was determined through plotting the relation (αhν)^2^ against energy (hν), as seen in Figure 10d–f. The band gap energy E_g_ of ZOA was calculated by drawing the tangent to the axis of energy to determine the optical band gap energy at (αhν)^2^ of 0. It showed 3.10 eV indicating a little shift from the band gap of pure ZnO because of the doping of aluminum inside the zinc oxide structure [7]. In the case of HZ-1-500, a large change was observed for the band gap energy because Figure 10e shows 2.60 eV. This strong effect of the nanohybrid structure was also observed for HZ-2-500 as shown in Figure 10f. Figure 10f revealed the narrowing of the band gap energy for HZ-2-500 to be 2.79 eV. The comparison with the pure zinc oxide showed strong narrowing for band gap energy because the reduction was from 3.30 eV to 2.60 and 2.79 eV for both the nanohybrids, indicating that the inorganic–magnetic-organic nanohybrids have a strong positive effect on the optical properties of zinc oxide.

### 3.5. Optical Activity

**Figure 11:**
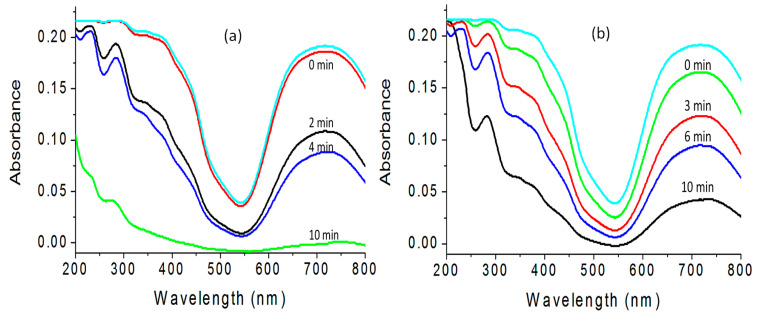
Photocatalytic degradation of naphthol green B in sunlight by: (**a**) HZ-1-500 and (**b**) HZ-2-500.

It is known that the improvement of the optical properties of the products of zinc oxides leads to positive effects for their photo activities. In order to indicate these positive effects, the prepared products have been used as photocatalysts to be appropriate means for increasing the photocatalytic activity of zinc oxide to decompose and remove pollutants by sunlight in short time. In this way, the green dye of naphthol green B was used to be specimen for industrial pollutants. The photo activities of zinc oxides (doped or undoped), and their products based on the nanohybrids structure were studied through photocatalytic degradation of naphthol green B. By measuring the absorbance of the liquid portion after exposure of the green solution of dyes to the sunlight for few minutes in the presence of the one of the prepared photocatalyst, the degradation of the main structure of the pollutant was observed through decreasing the intensity of the absorbance band at a wavelength of 714 nm, as seen in Figure 11a,b. At the same time, the reduction of the intensity of the absorption peaks at 322 nm, 280 nm, and 230 nm indicated the degradation of the naphthyl rings in the dye.

This blank experiment, which was performed without a photocatalyst, indicated the high stability of naphthol green B in sunlight. The photocatalytic degradation of the green dye was studied as a function of the time of sunlight exposure in the presence of the photocatalyst, as seen in Figure 11. When the aqueous solution of naphthol green B was mixed with the photocatalyst for 10 min in the dark, an appropriate change was observed, indicating that these photocatalysts have low adsorption power. For reference, it was used as 0 min irradiation.

Figure 11a showed the photo catalytic degradation of NGB under sunlight in the presence of HZ-1-500. By increasing the irradiation time, the photocatalytic degradation of naphthol green B increased. After 10 min of sunlight exposure, the green color was completely removed, indicating high activity for HZ-1-500. In the case of using HZ-2-500, the activity became lower, as shown in Figure 11b. The photocatalytic degradation of naphthol green B was arrived to 78% after 10 min of sunlight irradiation time. It means that the nanohybrid HZ-2-00 needs more than 10 min to completely remove the colored pollutant. It means that the nanohybrid HZ-1-500 is active and effective in sunlight because it completely destroyed the green dye at shorter time.

The high performance of the zinc oxide nanohybrids HZ-1-500 and HZ-2-500 was clear after comparison with the ZOA-500 and the pure zinc oxide. Where the complete removal of the green dye happened after 360 min of solar energy in presence of ZOA-500, in the case of the pure zinc oxide, the complete removal of the green dyes was achieved after 840 min of sunlight irradiation time. It means that the zinc oxide nanohybrids became very active in sunlight. In order to indicate the effect of the organic species on the optical activity, the kinetics of photocatalytic decolorization and degradation of naphthol green B were studied for both HZ-1-500 and HZ-2-500 through the next relation:

ln([C_o_]/[C]) = k × t (1)

The concentration of naphthol green B at certain times is coded as [C]. In the case of [C_o_], it represents the concentration of naphthol green B at t = 0. The rate reaction constant is k. To determine kinetically the type of reactions, ln([C_o_]/[C] was plotted in Y-axis against the irradiation time in minutes on the X-axis.

**Figure 12:**
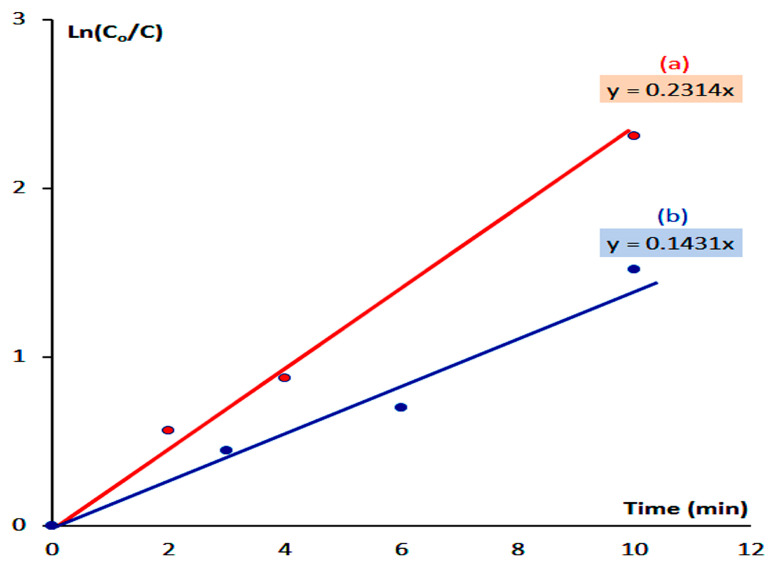
Kinetics study of the photocatalytic degradation of naphthol green B in sunlight by: (**a**) HZ-1-500 and (**b**) HZ-2-500.

Figure 12 shows a straight line indicating pseudo-first-order reactions for the reactions of photocatalytic degradation and decolorization of naphthol green B in the case of using both HZ-1-500 and HZ-2-500.

Figure 12b shows that the photo activity of HZ-2-500 led to the rate reaction constant of the photocatalytic degradation of naphthol green B in 0.143 min^−1^. By using HZ-1-500, Figure 12a indicates that the rate reaction constant increased to 0.294 min^−1^. The kinetics study concluded that the rate of photocatalytic degradation of naphthol green B in the presence of HZ-1-500 increased to be higher than that of HZ-2-500. It means that the low band gap of HZ-1-500 accelerated the photocatalytic degradation of naphthol green B. In addition, the zinc oxide nanohybrid, which was based on pure nanohybrid HZ-1 is better than the zinc oxide nanohybrid, which was produced from mixed phases between nanohybrid and nanolayered LDH.

### 3.6. Discussion

The fast photocatalytic degradation of the green dyes in sunlight showed the excellent activity of the prepared zinc oxide nanohybrid HZ-1-500 which was produced from inorganic-magnetic-organic nanohybrids. The high performance of HZ-1-500 can be explained through the novel approach for building the nanohybrid structure of HZ-1-500. The intercalation of the fine nanoparticles of CoFe_2_O_4_ nanocomposite among the nanolayers of Al/Zn created a good chance for incorporation of this nanocomposite with zinc oxide structures during the crystallization process. Therefore, HZ-1-500 has a good crystalline structure for zinc oxide and there are no peaks for aluminum or cobalt iron oxides. This good incorporation of CoFe_2_O_4_ nanocomposite with the crystals of zinc oxide partially failed for the sample HZ-2-500 because XRD results showed two mixed phases: nanolayered structure and nanohybrid. It means that the nanoparticles of CoFe_2_O_4_ nanocomposite could intercalate among the nanolayers of Al/Zn for part of the nanohybrid and support on the external surface of the plates of the nanolayered structure of Al/Zn. The good incorporation of CoFe_2_O_4_ nanocomposite with the crystals of zinc oxide which doped with aluminum created new optical active centers inside zinc oxide nanohybrid HZ-1-500 and caused reduction for its band gap energy to be very effective in sunlight because of the low band gap energy of CoFe_2_O_4_ (1.32 eV) [43]. At the same time, some sites of Zn in zinc oxide are occupied by CoFe2O4 atoms, producing new optical active centers called shallow traps between the valance band and conduction band, leading to decreasing for the band gap energy [1,44].

This low band gap energy and the small size of the nanoparticles of the zinc oxide nanohybrid HZ-1-500 have a strong effect on the mechanism of the photocatalytic degradation process of the green dyes. The mechanism of the photocatalytic degradation process is controlled by two critical reactions.

Photocatalyst + hν → h^+^(valance band) + e^−^(conduction band) (2)

Holes (h^+^) + H_2_O → H^+^ + ^•^OH (free radicals) (3)

Electrons (e^–^) + O_2_ → ^•^O_2_^−^ (4)

The first one depends on the amount of energy which was absorbed by the photocatalyst as seen in the Reaction (2). The second reaction is the movement and separation of light-induced electrons-holes, as shown in the Reactions (3) and (4). The photo-generated holes, which produced in the conduction band, react with the water molecules to produce highly oxidizing agents free radicals of hydroxyl groups (^•^OH). At the same time, the photo-generated electrons attack the oxygen molecules, which are adsorbed on the surface of the photocatalyst or dissolved in water to produce strong oxidizing agents superoxide radical anion (^•^O_2_^−^).

In the presence of green dyes, the molecules of NGB adsorbed on the surface of the nanoparticles of zinc oxide nanohybrid. The HZ-1-500 accelerated the first reaction in sunlight to become excited because it has absorbance from the wavelength 700 nm to 200 nm, in addition to low band gap energy as shown in the following Reaction (5).

HZ-NGB* (sunlight) → HZ + NGB^+^ intermediates + electrons (e^−^) (5)

Electrons (e^−^) + O_2_ → ^•^O_2_^−^ (6)

NGB+ intermediates + ^•^O_2_^−^ → Degradation compounds + CO_2_ + H_2_O (7)

The band gap of HZ-1-500 is not very small to accelerate the recombination reactions in addition to the shallow traps which help for separating between electrons and holes. Therefore, the degradation reaction continues as shown in Equations (6) and (7). By this way, the colored pollutants disappeared after ten minutes of sunlight exposure. For testing the re-use of the optimum sample, the photocatalytic degradation of HZ-1-500 was repeated two times for the fresh sample of the green dye. The same results were observed after 10 min of sunlight exposure, indicating high recyclability of the photocatalyst.

## 4. Conclusions

In the present study, a dual-aim was achieved for designing zinc oxide nanohybrids to be useful and effective for purifying water in sunlight. This aim focused on a new approach for building inorganic-magnetic-organic nanohybrids and producing effective zinc oxide nanohybrids in sunlight. In this line, two nanohybrids were prepared through expanding the nanolayered structures of Al/Zn by intercalating long chains of hydrocarbons of fatty acids such as n-capric and stearic acid to facilitate the insertion of very fine nanoparticles of cobalt iron oxides among the nanolayers of Al/Zn. The characterization techniques showed that the prepared inorganic-magnetic-organic nanohybrids were useful for producing zinc oxide nanohybrids by thermal treatment. By measuring the optical properties, a clear reduction of band gap energy was observed for the prepared zinc oxide nanohybrids compared with the doped and undoped zinc oxide. This reduction of band gap energy from 3.20 eV to 2.60 eV led to high activity for the prepared zinc oxide nanohybrids in sunlight.

This high activity was proven by a complete removal of naphthol green B after 10 min of sunlight exposure in presence of the prepared zinc oxide nanohybrids. These results were confirmed by the comparison with the pure and doped zinc oxide which indicated that the pure and doped zinc oxide removed the green dyes after 360–840 min of sunlight exposure. Furthermore, the kinetic study showed that the zinc oxide nanohybrid, which was based on the pure nanohybrid, is better than the zinc oxide nanohybrid, which was produced from mixed phases between the nanohybrid and nanolayered LDH. Finally, it can be concluded that this new approach for designing photoactive nanohybrids led to positive tools for facing energy- and water-related problems through using renewable and non-polluting energy for purifying water.

## Data Availability

Data available in a publicly accessible repository.
